# *In Vivo* Mapping of the Choriocapillaris in High myopia: a Widefield Swept Source Optical Coherence Tomography Angiography

**DOI:** 10.1038/s41598-019-55192-w

**Published:** 2019-12-12

**Authors:** Rodolfo Mastropasqua, Pasquale Viggiano, Enrico Borrelli, Federica Evangelista, Daniele Libertini, Luca Di Antonio, Lisa Toto

**Affiliations:** 1 Ophthalmology Clinic, Department of Medicine and Science of Ageing, University G. D’Annunzio Chieti-Pescara, Chieti, Italy; 2 Bristol Eye Hospital, Bristol, UK

**Keywords:** Cells, Epidemiology, Preclinical research

## Abstract

To report variation of choriocapillaris (CC) flow in widefield in high in myopic subjects compared with an age-matched normal control group using ultra widefield optical coherence tomography angiography (UW-OCTA). This is a Prospective, cross-sectional study. Thirty high myopia subjects and fifty healthy subjects were enrolled. Healthy and high myopia subjects were imaged with the SS-OCTA system (PLEX Elite 9000, Carl Zeiss Meditec Inc., Dublin, CA, USA). For each eye, five 12 × 12-mm OCTA volume scans were acquired. The *en face* CC images were then exported to imageJ and a semi-automated algorithm was used for subsequent quantitative analysis. The main outcome was a quantitative analysis of the CC. This analysis was performed in three different regions: (i) peripapillary, (ii) macular, and (iii) periphery. In addition, CC variables were further investigated in distinct fields within these three different regions. Thirty myopic eyes (32 subjects; myopic group) and fifty eyes (50 subjects; control group) without elevated myopia were included in the analysis. Mean ± SD age was 26.9 ± 2.9 years [median: 27 years; range: 20.0–40.0 years]. Mean ± SD axial length was 26.6 ± 0.6 mm [median: 26.2 mm; range: 26.1 to 28.0 mm]. Mean ± SD axial length was 26.6 ± 0.6 mm [median: 26.2 mm; range: 26.1 to 28.0 mm] in the myopic group and 23.9 ± 1.1 mm [median: 23.9 mm; range: 21.8 to 25.9 mm] in the control group. The total signal void area was significantly greater in myopic eyes compared with control group. The peripapillary region exhibited the greatest total signal void area (p < 0.0001 vs macular region, p < 0.0001 vs peripheral region). Within the macular region, the foveal area exhibited a greater total signal void area in comparison with both the parafoveal area (p < 0.0001) and the perifoveal area (p < 0.0001). In conclusion we report quantitative mapping of the choriocapillaris in myopic eyes compared with an age-matched normal control group. The CC perfusion appears to have a wide topographical variation.

## Introduction

The choriocapillaris (CC) represents an approximately 10-μm-thin layer of capillaries limited to the innermost part of the choroid, located between Bruch’s membrane and the medium-sized choroidal vessels^1^. Histological evidences suggest that this thin vascular layer is organized into a series of hexagonal-shaped lobules, which are smaller at the posterior pole and become progressively larger toward the periphery^1,2^. Furthermore, these tiny capillaries are characteristically separated by many small inter-capillary spaces that are wider and progressively more elongated towards the retinal periphery^2^. The development of optical coherence tomography angiography (OCTA) has significantly improved our capability to visualize and investigate the CC thanks to greater sensitivity and the use of a longer wavelength which better penetrates deeper tissues and is less scattered by the RPE. Importantly, a higher speed system allowed the acquisition of a larger field of view, ensuring a wider investigation of the retinal and choroidal vessels. Therefore, this kind of technology grants to image from the macula to the far retinal periphery and is thus termed ultra-widefield (UWF) OCTA imaging^3^. [Borrelli E, Viggiano P, Evangelista F, Toto L, Mastropasqua R. Eyelashes Artifact in Ultra-Widefield Optical Coherence Tomography Angiography. Ophthalmic Surg Lasers Imaging Retina. In press].

Using OCTA, several studies have demonstrated that the CC assessment was characterized by topographical variability correlating with age and ocular or systemic disease (hypertension, high myopia, age-related macular degeneration)^1,4–8^.

Myopia is one of the leading causes of visual impairment in many developed countries. In high myopia the increased axial length may lead to development of several retinal complications, including posterior staphyloma, retinoschisis, lacquer crack (LC) formation, chorioretinal atrophy, and myopic neovascularization (mNV)^9,10^. Previous studies using different imaging modalities have investigated choroidal blood flow changes showing a decreased total number of flow voids CC associated with high myopia^5,11,12^. These studies have improved the evaluation on the CC function and characteristics, however an important limitation was that this assessment was limited to the macular region.

The introduction of UWF OCTA imaging allowed new insights into the choroidal microvasculature beyond the posterior pole. A wider investigation of the CC might provide more details about the choroidal disorders in high myopia not limited to the macula. The UWF-OCTA imaging has thus opened the door for a better understanding to the diagnosis and prognosis of different retinal and choroidal conditions.

The aim of this study was to show the difference of the CC flow in widefield in high myopic eyes and non-high myopic eyes using UWF OCTA imaging. Notably, these measurements were determined in different retinal regions in order to provide a topographical analysis of the CC perfusion.

## Methods

### Study participants

This is a prospective observational cross-sectional study, subjects between 18 and 40 years of age with high myopia (axial lengths longer than 26 mm) without any structural changes, including post pole changes like Dome shaped macula, myopic CNV or myopic posterior staphyloma were enrolled at the ophthalmology clinic of University G. d’Annunzio, Chieti-Pescara, Italy. Age-matched healthy individuals without elevated myopia, without any visual symptoms and without any history of previous ocular or systemic diseases were eligible for this study. We enrolled only young patients except children because UWF OCTA needs clear lens and good visual fixation. The study was approved by the Institutional Review Board (IRB) (Department of Medicine and Science of Ageing, University G. d’Annunzio Chieti-Pescara) and adhered to the tenets of the Declaration of Helsinki. An Institutional Review Board approved informed consent was obtained from all patients.

All subjects enrolled were imaged with the PLEX Elite 9000 device between April 2018 and July 2018. Moreover, all patients received a complete ophthalmologic examination, which included the measurement of best-corrected visual acuity (BCVA), intraocular pressure (IOP), and dilated ophthalmoscopy. Exclusion criteria were: (i) evidence or history of ocular diseases; (ii) evidence or history of systemic disorders, including diabetes and systemic hypertension; (iii) history of previous ocular surgery; (iv) eyes with diffuse RPE atrophy due to high myopia or any structural changes, including myopic CNV or myopic posterior staphyloma;

### Imaging

Subjects underwent OCTA imaging using the PLEX Elite 9000 device (Carl Zeiss Meditec Inc., Dublin, CA, USA) which uses a swept laser source with a central wavelength of 1050 nm (1000–1100 nm full bandwidth) and operates at 100,000 A-scans per second.

For each eye, five 12 × 12-mm OCTA volume scans were acquired. These scans were acquired in five different patient’s gazes (central, nasal inferior, nasal superior, temporal inferior, and temporal superior) by moving the internal fixation light. For each eye we thus obtained five OCTA scans from five distinct and partially overlapping retinal regions. FastTrac motion correction software was used while the images were acquired.

Subjects underwent IOL Master (Carl Zeiss Meditec AG, Jena, Germany) for ocular biometry, including AL and keratometry measurements.

Poor quality images (signal strength index (SSI) < 8) with either significant motion artifact or incorrect segmentation were excluded.

For all the participants, the best quality image among 3 acquired images of the most myopic eye from each subject was selected to be analyzed in the study.

All selected images were carefully visualized by two retinal specialists (RM and PV) in consensus to ascertain the correctness of segmentation and in case of erroneous recognition by the software of the position of the boundaries of the inner limiting membrane (ILM) and retinal pigment epithelium (RPE) manual correction was performed using the segmentation and propagation editing tool from the device.

### Image processing

The main outcome measure was the total signal void area, which represents a measure of the total area of CC vascular dropout (absence of flow or flow below the slowest detectable threshold).

In order to quantify these variables, a slightly modification of a previously reported algorithm was employed^4,6–8,13,14^. In brief, for each eye, we first exported the superficial capillary plexus (SCP) and CC *en face* OCTA images (resolution of 500 × 500 pixels), which were automatically segmented by PLEX Elite 9000 device. These images were then imported in ImageJ software version 1.50 (National Institutes of Health, Bethesda, MD; available at http://rsb.info.nih.gov/ij/index.html) and, consequently, the Phansalkar method was used to binarize the CC images. Obtained images were processed with the ‘Analyze Particles’ command, in order to assess the total signal void area and to count and measure the signal voids. The CC directly beneath major superficial retinal vessels was excluded from analysis to eliminate potentially confounding shadow or projection artifacts.

**Figure 1:**
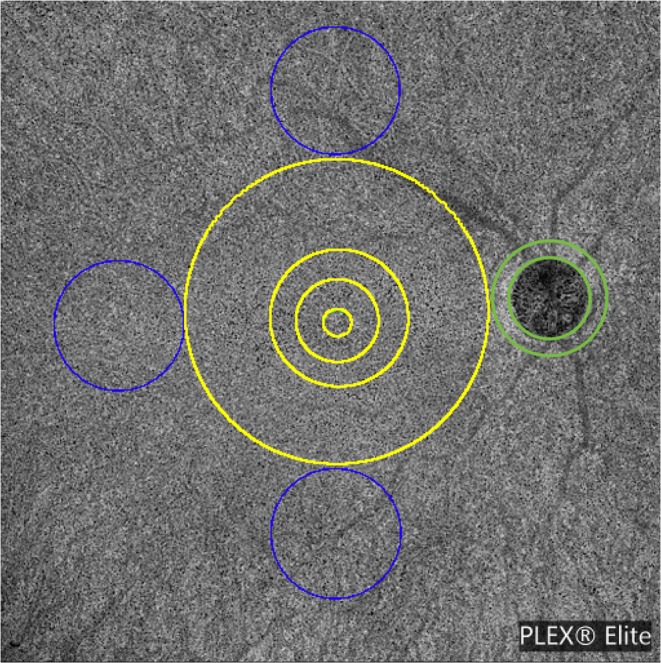
Representation of the regions used to investigate optical coherence tomography angiography variables. OCT angiography variables were investigated in three different regions of the choriocapillaris: (i) *macular region* (yellow circle) with a diameter of 6 mm; (ii) *peripapillary region* which consists in a 500-μm-wide annulus around the optic disc (green annulus), and (iii) *periphery region* which was assessed in three circles tangential to the macula (blue circles) and with diameters of 4.5 mm. The macular region was further divided into the foveal area (diameter of 1.5 mm), the parafoveal area (diameter of 2.5 mm), and the perifoveal area (diameter of 6 mm). The periphery region was separately investigated in the superior, temporal, and inferior areas (blue circles).

The quantitative analysis was performed in three different regions: (i) *macular region*, which was defined as a circle centered on the fovea with diameter of 6 mm, (ii) *peripapillary region* which consists in a 500-μm-wide ring around the optic disc, and (iii) *periphery region* which was assessed in three circles tangential to the macula and with diameters of 4.5 mm (Fig. 1)^15^. The latter choice was made to investigate the CC in the near and mid periphery (1.5-mm-wide annulus around the macula and 3.0-mm-wide annulus around the near periphery, respectively) and consequently to exclude the far periphery from the analysis.

Furthermore, the analysis of the macular and periphery regions was further performed in different sub-fields: the macular region was further divided into the foveal, parafoveal and perifoveal areas (with diameters of 1.5 mm, 2.5 mm, and 6 mm, respectively)^15^, while the periphery was split into the superior, temporal and inferior areas (Fig. 1).

In order to assess reproducibility of our measurements, 15 healthy patients underwent second OCTA measurements in a different day.

### Statistical analysis

All quantitative variables were reported as mean and median and standard deviation or interquartile range (IQR) in the Results section and in the tables.

To detect departures from normality distribution, Shapiro-Wilk’s test was performed for all variables. Since CC variables did not show a normal distribution, non-parametric Mann-Whitney U test was conducted to investigate differences in quantitative CC variables between the two groups. The analysis of variance was used to assess the reproducibility between the two measurements. In order to assess reproducibility of our measurements, 15 high myopic patients underwent second OCTA measurements in a different day. Spearman’s correlation coefficient was tested to evaluate the linear correlation between CC perfusion and choroidal thickness in myopic eyes.

Statistical calculations were performed using Statistical Package for Social Sciences (version 20.0, SPSS Inc., Chicago, IL, USA). The chosen level of statistical significance was p < 0.05.

The sample size of the study was tested to be proper for a mean difference between groups of almost 10%, a power of 80% and type I error rate (α) of 5%.

## Results

### Characteristics of subjects included in the analysis

Of the 32 eyes (32 individuals) that were initially enrolled, 30 eyes met the required image quality criteria and were used in the analysis.

Thirty eyes of 30 patients (20 female, 10 male) with high myopia were included in this study (myopic group). Mean ± SD age was 26.9 ± 2.9 years [median: 27 years; range: 20.0–40.0 years]. Mean ± SD axial length was 26.6 ± 0.6 mm [median: 26.2 mm; range: 26.1 to 28.0 mm].

Fifty eyes from 50 subjects without elevated myopia (20 males, 30 females) were also included in the analysis (control group). Mean ± SD age was 25.2 ± 5.1 years [median: 24.5 years; range: 20.0–40.0 years]. Mean ± SD axial length was 23.9 ± 1.1 mm [median: 23.9 mm; range: 21.8 to 25.9 mm]. All myopic and control subjects had a visual acuity of 20/20 Snellen.

### Widefield OCTA analysis of the choriocapillaris

The difference between the 2 measurements was not statistically significant in all the analyzed regions (P > 0.05), indicating an overall good reproducibility.

The total signal void area was significantly greater in myopic eyes compared with control group. Within the macular region, the foveal area exhibited a greater total signal void area (median: 22.5% and IQR 20.8–24.6%), in comparison with both the parafoveal area and the perifoveal area (p < 0.0001) in both groups (Table 1).

**Table 1:** Tested choriocapillaris optical coherence tomography variables in control and myopic group.

	Control Group	Myopic group	P value
CC fovea Total signal void area (%)	14.7 (12.0–16.5)	22.5 (20.8–24.6)	<0.001
CC parafovea Total signal void area (%)	13.1 (11.3–15.9)	21.2 (19.7–22.5)	<0.001
CC perifovea Total signal void area (%)	13.0 (10.8–15.9)	21.0 (19.3–22.8)	<0.001

Data are presented as median (interquartile range); CC: choriocapillaris.

Using nonparametric test, Mann-Whitney U test.

The peripapillary region in myopic group was characterized by an higher total signal void area, as compared with age-matched control group; (median: 24.3% and IQR 22.4–27.3% in the myopic group; median: 16.7% and IQR: 13.5–19.2% in the control group) (p < 0.0001) (Tables 2 and 3).

**Table 2:** Tested choriocapillaris optical coherence tomography variables in control and myopic group.

	Control Group	Myopic group	P value
CC paraON Total signal void area (%)	16.7 (13.5–19.2)	24.3 (22.4–27.3)	<0.001

Data are presented as median (interquartile range); CC: choriocapillaris; paraON: optic nerve.

Using nonparametric test, Mann-Whitney U test.

**Table 3:** Choriocapillaris OCTA tested variables in the analyzed regions in myopic eyes.

	Macula	Periphery	Peripapillary
Total Signal Void Area (%)	21.6 (19.9–22.8)	22.6 (20.9–24.7)	24.3 (22.4–27.3)
		<0.0001^a^	<0.0001^a^
	—		<0.0001^b^

Data are presented as median (interquartile range).

^a,b^Mann-Whitney U test ^a^comparison versus “Macular region”; ^b^comparison versus “Periphery region”.

In the analysis investigating the near/mid periphery region, we also found an increased ischemia of myopic eyes in all regions. In particular, the superior and inferior sectors were characterized by an higher total signal void area, as compared with control group (median: 23.6% and IQR 21.6–25.1% in the superior region; median: 23.3% and IQR: 21.6–24.4% in the inferior region in myopic eyes) (median: 12.9% and IQR 11.6–15.1% in the superior region; median: 13.2% and IQR: 11.2–16.1% in the inferior region in control group) (p < 0.0001) (Table 4).

**Table 4:** Tested choriocapillaris optical coherence tomography variables in control and myopic group.

Near/Mid Periphery	Control Group	Myopic group	P value
CC Superior Total signal void area (%)	12.9 (11.6–15.1)	23.6 (21.6–25.1)	<0.001
CC Inferior Total signal void area (%)	13.2 (11.2–16.1)	23.3 (21.6–24.4)	<0.001
CC Temporal Total signal void area (%)	12.3 (11.0–14.3)	20.9 (19.7–23.2)	<0.001

Data are presented as median (interquartile range); CC: choriocapillaris.

Using nonparametric test, Mann-Whitney U test.

In myopic eyes, we did not find significant correlations between CC perfusion and choroidal thickness in the analyzed sectors (Rho = −0.328 and p = 0.136 for the foveal region, Rho = −0.332 and p = 0.132 for the parafoveal region, Rho = −0.311 and p = 0.159 for the perifoveal region, Rho = −0.333 and p = 0.130 for the peripheral superior region, Rho = 0.296 and p = 0.181 for the peripheral inferior region, Rho = 0.072 and p = 0.751 for the peripheral temporal region).

## Discussion

In this cross-sectional study, we report quantitative mapping of the choriocapillaris in myopic eyes versus a healthy age-matched control group using widefield SS-OCTA. We found regional differences in the CC perfusion which should be taken into account as OCTA–based parameters are increasingly used for the diagnosis of choroidal vascular disorders in adult eyes. This study demonstrated a reduced perfusion in the CC in the myopic group versus the age-matched healthy cohort. Furthermore, a topographical analysis revealed that both the myopic and control group CC does not exhibit a uniform perfusion. Importantly, in the assessment of the CC in the retinal periphery, we excluded the far periphery from the evaluation. This assessment is indeed still limited by significant shadowing artifacts, given that the very large depth of field commonly results in the patient’s eyelashes to appear in the image [Borrelli E, Viggiano P, Evangelista F, Toto L, Mastropasqua R. Eyelashes Artifact in Ultra-Widefield Optical Coherence Tomography Angiography. Ophthalmic Surg Lasers Imaging Retina. In press.]. Thus, we felt the safer strategy was to look just at the near/mid periphery.

Previous notable OCTA studies on the CC were limited in assessing the macular region^1^ and showed a correlation with age and axial length, as assessed in both pediatric and adult cohorts^4,5,16^. In particular, using OCTA, the CC perfusion seems to be reduced in the foveal area, as compared to the parafoveal and perifoveal regions^5,17^. Consistently to previous reports we found topographical differences within the macular region, since the CC directly beneath the fovea was characterized by a lower perfusion in both groups although myopic eyes presented an increase of the whole macular total signal void area versus the control group (Table 1). This finding confirmed previous works highlighting how the myopic eyes are characterized by a lower CC perfusion in comparison with the age-matched healthy cohort^5,11,18,19^. Hirata and Negi^11^ reported a reduced CC perfusion in myopic eyes suggesting that this finding might be related to the decrease in CC density to capillary thinning observed with transmission electron microscopy and partly to overall stretch of the capillary network caused by abnormal enlargement of the myopic eyes^11,18,19^.

Ultrastructural evidence has identified that the CC origins from larger diameter vessels that are supplied by the short posterior ciliary arteries (SPCAs) and long PCAs (LPCAs)^1^. The SPCAs invade the eye figuring a circle around the optic disc whereas the LPCAs supply the region extending from the macula to the periphery^20–22^. As with previous studies^5,18,19^ the decrease choroidal blood flow in degenerative myopia was attributed to increased vascular resistance and narrowing in the posterior ciliary artery. In addition, it has been also speculated that the choroid might be divided in specialized and distinct zones, which include the peripapillary zone, the region underlying the macula, and the periphery. Using widefield SS-OCTA we explored these choroidal distinct zones in myopic and control group adding to the literature quantitative data of the CC.

Myopic eyes in our study did show a significantly increase signal void area compared with an age-matched normal control group in all regions. The latter finding is in agreement with previous histopathological studies illustrating that the area of flow deficit in the CC is increased in eyes with greater myopia^5,11,12,18,19,23,24^. However, a further topographical sub-analysis revealed that the peripapillary zone was actually characterized by an increase of the signal voids, as compared with the CC distinct zones in myopic group (Table 3). In agreement with previous studies^25,26^, we hypothesized that this finding could be secondary to the expansion of the globe leading to a localized deformation of the peripapillary region with CC stretch. Thus, it is possible that the reduction of the choriocapillaris network as visualized by SS-OCTA could be a direct consequence of this mechanical deformation. We cannot, however, exclude the possibility that these alterations in the peripapillary CC area might be the result of early atrophy in eyes with no clinically apparent atrophy spreading further light on the CC function in myopic eyes^23^.

Recent studies of highly myopic eyes using UWF reported numerous abnormalities of the retinal vasculature in the far periphery such as retinal capillary telangiectasia and retinal capillary microaneurysms^27,28^. In our adult cohort, we also assessed flow characteristics within the CC periphery and we noted an increased ischemia of myopic eyes (Table 4). Future studies investigating the retinal periphery might provide additional information regarding different myopic chorioretinal alterations.

Our study has several limitations which should be considered when interpreting our results. First, the 12 × 12-mm scan has limited sampling density with a larger sampling space (24 micron) that is larger than the system lateral resolution (14 micron). Therefore, the CC assessment may be severely down-sampled. Second, while we excluded the far peripheral regions from the analysis in order to avoid eyelashes artifacts from confounding the quantitative assessment, we are not able to exclude that these artifacts may have impacted, at least in part, also the investigation of the inferior and superior near/mid periphery regions. In addition, the OCTA technique used in this study has limitations including motion and projection or shadowing artifacts. As an example, the superficial retinal vessels can still confound the analysis of the CC. However, we did use a longer wavelength to image the CC and we employed an image processing to mask region beneath major superficial vessels. Of note, we are not able to comment on the magnification effect (distortion) secondary to the elongated shape of eye globe which might have occurred in our images. Finally, our study includes only healthy young subjects, therefore, we are unable to comment upon the effect of the age on widefield CC perfusion in myopic eyes.

In summary, in this widefield SS-OCTA study of the CC, we observed the CC flow characteristics in myopic eyes versus a healthy age-matched control. Future studies with extended longitudinal follow up might explain the increased CC ischemia in myopic eyes providing additional substantive information on the role of CC layer.
